# Supplementary material for: Deepprune: Learning Efficient and Interpretable Convolutional Networks Through Weight Pruning for Predicting DNA-Protein Binding
Source: Front Genet. 2019 Nov 20;10:1145. doi: 10.3389/fgene.2019.01145 (PMC6879555; doi:10.3389/fgene.2019.01145)
Supplement: Supplementary file 1 [file DataSheet_1.pdf]

Supplementary Material

1 SUPPLEMENTARY FIGURES

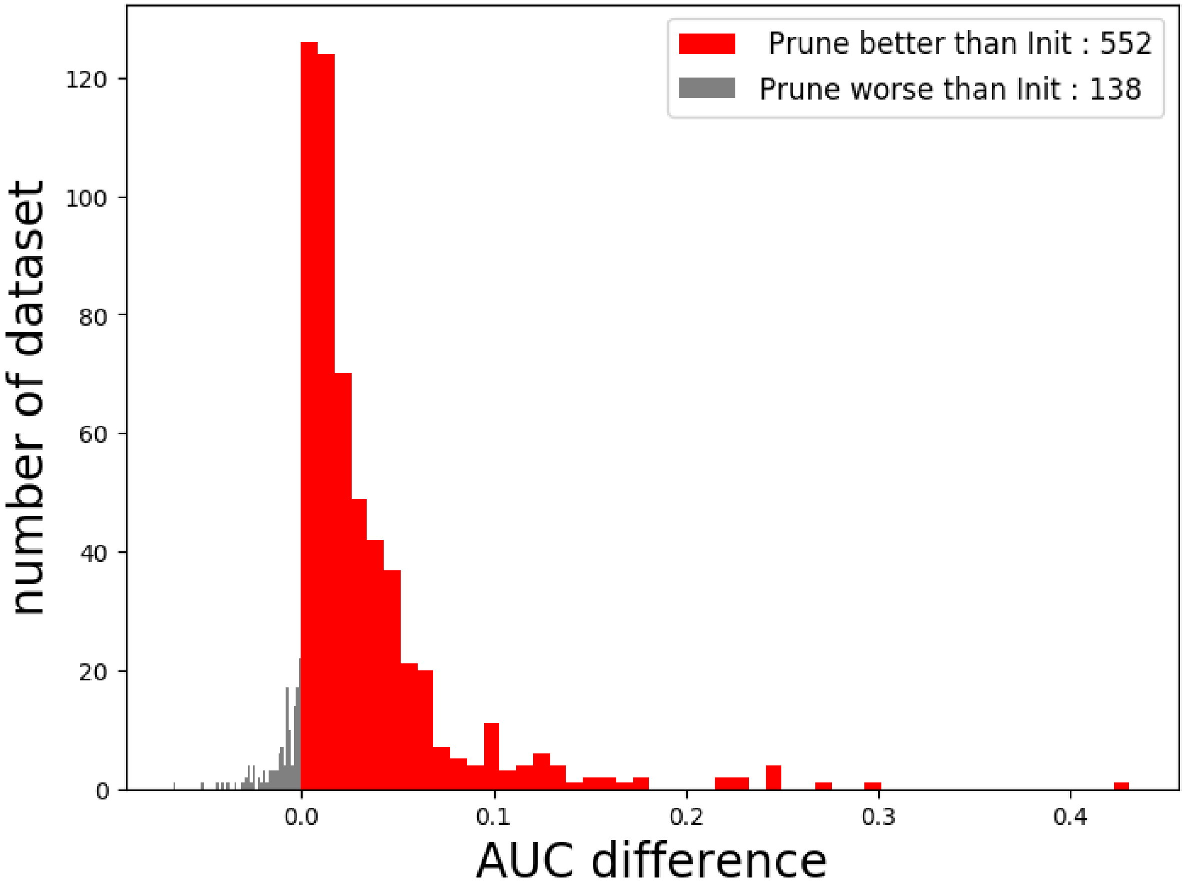

Figure S1. The performance of Deepprune with reinitialization of winning tickets on real datasets where kernel length = 15 and kernel number = 4. Deepprune with reinitialization of winning tickets increases the AUC on real datasets. The x axis shows the AUC difference under the baseline (Init) and Deepprune (Prune). Deepprune with re-initialization is better than baseline on 552 datasets, but worse than baseline with 138 datasets. This figure clearly shows that Deepprune with reinitialization of winning tickets achieves better performance with limited kernel number.

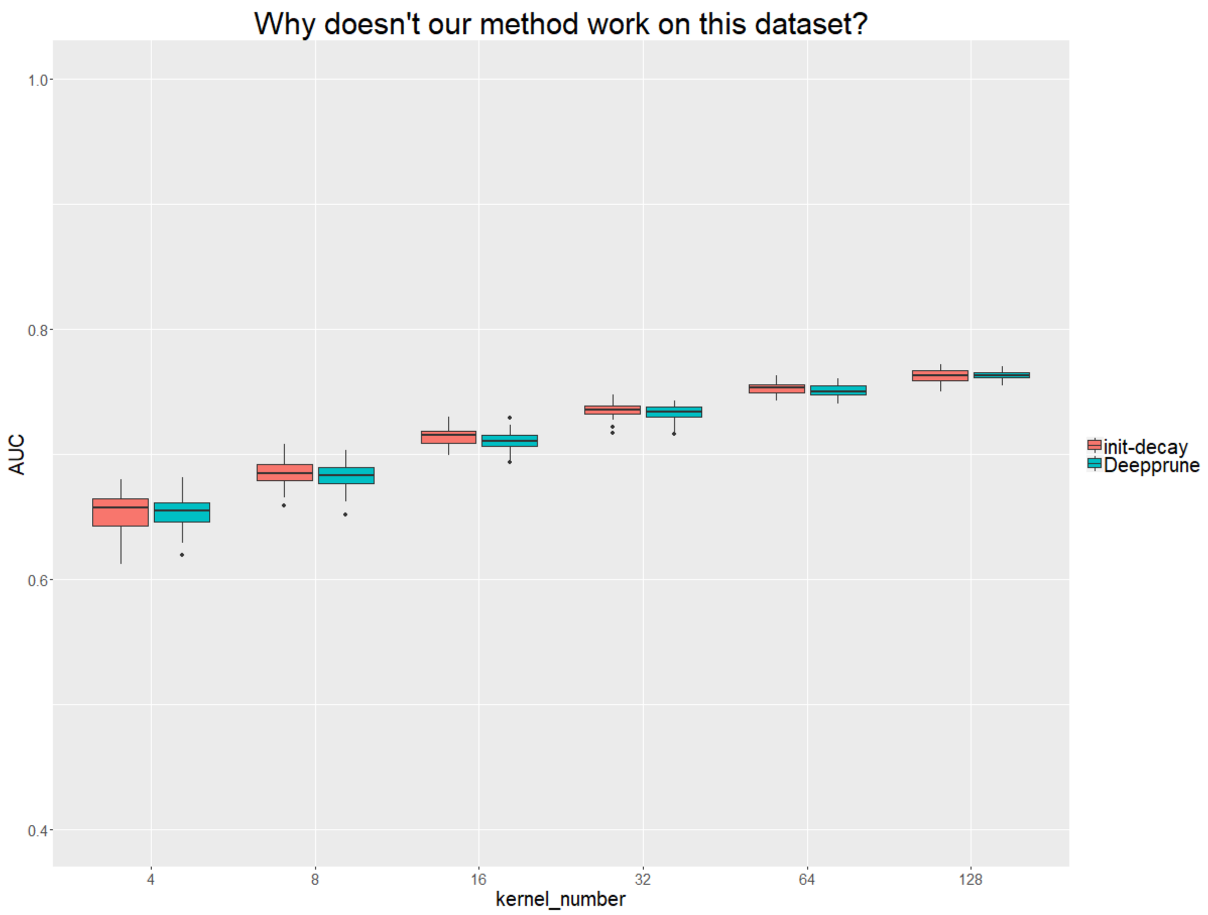

Figure S2. We selected the datasets in which the performance of Deepprune was below 0.01 and executed them 10 times with different random seeds to determine whether the poor performance was consistent. As shown, on these datasets, Deepprune is not always worse than the baseline; in fact, the mean performance of the two models is almost identical. However, we also found that our model performances worse in part of the datasets. We suspect that the underlying model of these datasets is complex.

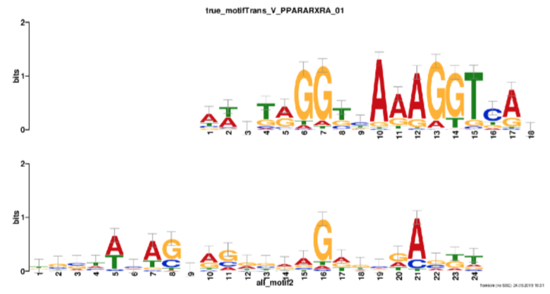

(a) The best recovered motif of the baseline

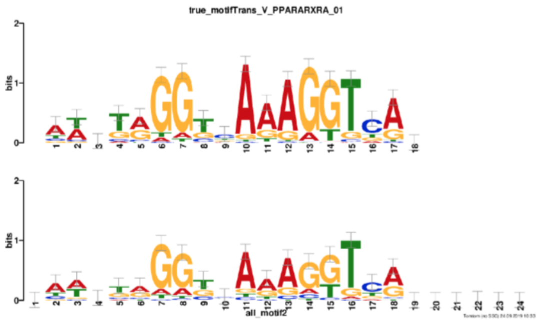

(b) The best recovered motif of Deepprune(long motif)

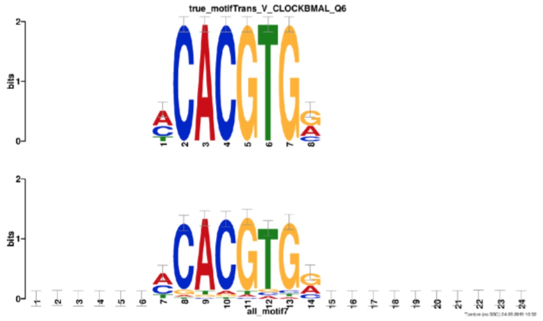

(c) The best recovered motif of Deepprune(short motif)

Figure S3. Motifs recovered by our model (the last two) and by baseline (the first) aligned to the true given motifs (top row) in the case of 8 kernels. Utilizing Tomtom algorithm, the E-values of the motifs recovered by Deepprune are 7.59×10^{-23}, 2.00×10^{-7}, respectively. At the same time, the ones recovered by baseline are 5.27×10^{-2}.

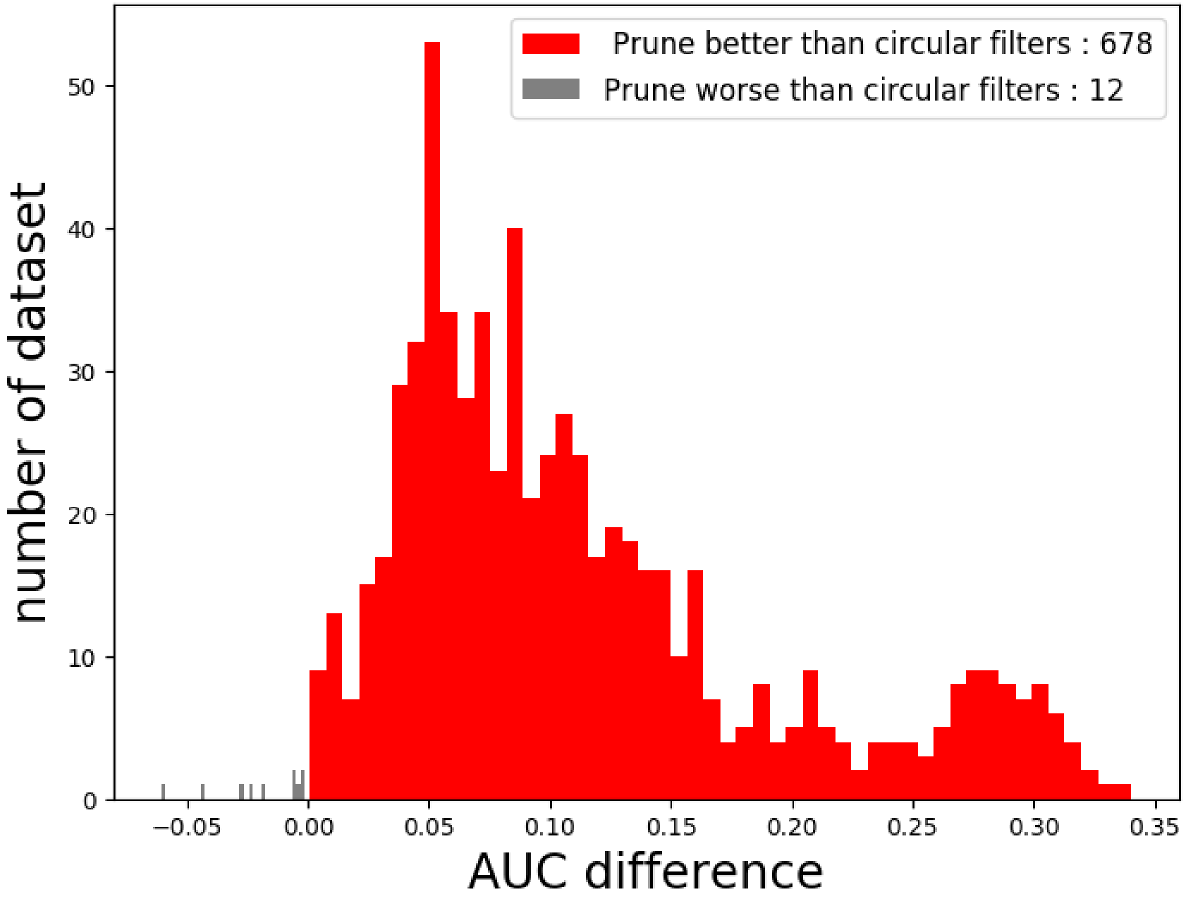

Figure S4. The performance of Deepprune compared with circular filters on real datasets where kernel length = 15 and kernel number = 4. Deepprune outperforms circular filters on real datasets. The x axis shows the AUC difference under the circular filters and Deepprune (Prune). Deepprune is better than circular filters on 678 datasets, but worse with 12 datasets. This figure clearly shows that Deepprune achieves better performance with limited kernel number.

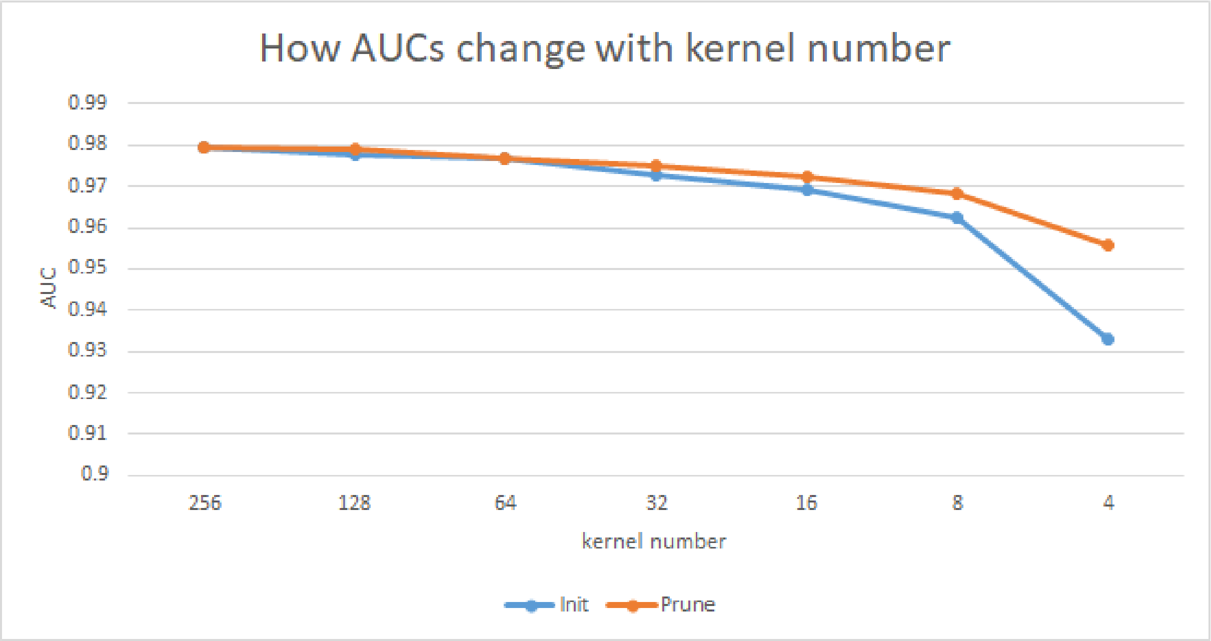

Figure S5. The prediction accuracy of the baseline(Init) and Deepprune(Prune) versus the number of kernels for real data example 1.

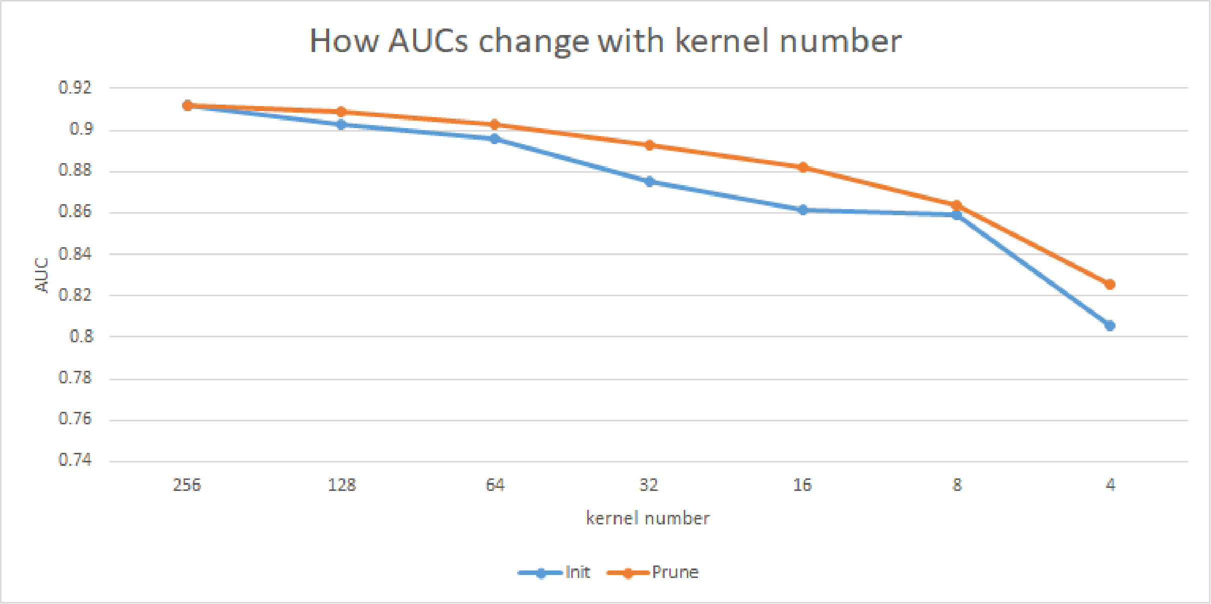

Figure S6. The prediction accuracy of the baseline(Init) and Deepprune(Prune) versus the number of kernels for real data example 2
